# Reviews

**Published:** 1880-11

**Authors:** 


### Reviews.

**A Treatise on the Practice of Medicine.** For the use of Students and Practitioners. By Robert Bartholow, M. A., M. D., LL. D., Prof. of Materia Medica and General Therapeutics in the Jefferson Medical College of Philadelphia, etc. New York: D. Appleton & Co., 1, 3 and 5 Bond street. 1880.

This work of 850 pages octavo, is one that we can honestly and do most heartily recommend. The writer is well known as the author of a work upon Materia Medica and Therapeutics, one highly valued, but not more justly so than the present treatise. The author has had a large experience in the study and treatment of disease, and this is his apology for adding another to the already long list of text-books upon practice.

In his preface he reviews the special privileges enjoyed by himself in the study of disease.

"Serving as an officer of the Medical Staff of the United States Army in Kansas, Utah, Colorado, New Mexico, Minnesota, and during the war of the rebellion at Washington, Nashville, Chatanooga, Baltimore, etc., followed by an extensive practice (private and hospital) of sixteen years, at Cincinnati, I may justly claim to have enjoyed large opportunities for the clinical study of the diseases of the North American Continent. With one or two unimportant exceptions, I have had personal charge of the maladies treated of in this work, and have made them the subject of clinical demonstration and *post mortem* investigation, either privately or in public lectures."

While being well pleased with the work in every respect, we wish particularly to indicate our appreciation of the author's views upon treatment. He says: "The reader will find that I have no sympathy with the therapeutical nihilism of the day. * * Indeed there is no department of the subject in which it seemed to me so necessary to express positive opinions." Another great advantage is found in the fact that the later discoveries in Pathology have been incorporated.

In this respect the treatise is fully up to date and very complete. Both to student and practitioner we once more strongly and unreservedly recommend this work.

---

**The Transactions of the Medical Association of the State of Missouri.**

This is not a large volume, but contains among its papers one or two of special merit. The president, in his address, on "Medical Ultraisms" indulges in some hard hits at the expense of the gynæcologists, but in general it is marked by strong common sense views. From the report of the committee on medical education, it appears the schools in that portion of the country are being subjected to healthy criticisms, which in some instances, at least, cannot but prove beneficial.

**Atlas of Skin Diseases.** By LOUIS A. DUHRING, M. D, Professor of Skin Diseases in the Hospital of the University of Pennsylvania, &c. Part vii. Philadelphia: J. B. Lippincott & Co. 1880.

We have, in a previous number of the Journal, directed attention of the profession to this valuable atlas, designed by Prof. Duhring, to assist in the study of this obscure and difficult class of diseases. Part vii is of equal value to those that have preceded it, and gives large and accurate illustrations of Exzema Pustulosum, Impetigo Contagiosa, Syphiloderma Papulosum and Lupus Vulgaris. The text accompanying each plate gives a clinical lecture upon the disease illustrated in the plate. The work is designed to be a clinical digest in Dermatology. The plan is an excellent one, and the manner in which it has been executed thus far deserves, as it has already received, the highest encomiums of the profession. We shall look with interest for the succeeding numbers. The work, when completed, will constitute the most practical treatise upon skin diseases yet offered to the profession.

---

**A Practical Treatise on Tumors of the Mammary Gland; embracing their Histology, Pathology, Diagnosis and Treatment.** By SAMUEL W. GROSS, A. M., M. D., Surgeon to and Lecturer on Clinical Surgery in the Jefferson Medical College Hospital and the Philadelphia Hospital. New York: D. Appleton & Co.

Workers with the microscope now know how to use it, and are better able than ever to read rightly its teachings. They are started on the right road, and have already established many undisputed facts. So true is this of histology and pathology, that with a feeling of security never known before, investigators are speaking with authority, and are bringing their labors into a close test with clinical experience. Dr. Gross is the son of the well known surgeon, Samuel D. Gross, and to him this able monograph of 250 pages is dedicated. The object of the volume is to "reconcile clinical features and the minute structure of tumors of the mammary gland," according to the light of modern histological research. The deductions are founded upon the clinical study "of sixty-five cases of cysts, and nine hundred and two neoplasms, the nature of which has been confirmed by the microscope, and more than one-seventh of which are original." The work is at once original, scientific and practical. Its histology will receive the careful attention of the specialists; the surgeon will find it a guide and help; while the general practitioner, who would be informed, and desires to know what will be taught in our text-books and in our colleges, must study it. It is an effort that will add new honor to an honorable name, and of which we, as Americans, may all be proud as an example of original work and investigation.

---

**A Manual of Minor Surgery and Bandaging.** By CHRISTOPHER HEATH, F. R. C. S., Surgery to University Hospital, etc. Sixth edition, revised and enlarged. With one hundred and fifteen illustrations. Philadelphia: Lindsay & Blakiston. 1880.

The author is eminently a writer for students, and as such he is very successful. The present little book is written especially for young house-surgeons, and the author's aim has been to describe the treatment of the various accidents and emergencies coming under their care, and to call attention to those minor points, which are generally imparted only by oral instructions. Although written more especially for English house-surgeons, the duties and responsibilities of which are minutely described, it may be studied with advantage and pleasure by American students and young surgeons.

---

**A New School Physiology.** By RICHARD DUNGLISON, A. M., M. D., author of The Practitioner's Reference Book, editor of Dunglison's Medical Dictionary, &c., &c. Illustrated with 117 engravings. Philadelphia: Porter & Coates.

A knowledge of the general structure of the body is most important to all young people and should be taught generally in our public schools. One of the best, if not the best text book on Physiology for such a purpose, is this new work of Dr. Dunglison. The publishers deserve credit for excellent press-work, and the illustrations are very good.

**Health and Healthy Homes. A Guide to Domestic Hygiene.** By GEORGE WILSON, A. M., M. D., Medical Officer, &c. With notes and additions by J. G. RICHARDSON, M. D., Professor of Hygiene in the University of Pennsylvania. Philadelphia: Presley Blakiston, 1012 Walnut street. 1880.

This work of 304 pages designs to be a trustworthy guide to Domestic Hygiene in all its branches. The author discusses the structure and function of the human body; the causes of disease; food and diet; cleanliness and clothing; exercise, recreation and training; the home and its surroundings; infectious diseases and their prevention. It will be seen that he has selected subjects of great practical interest and value to every household, and the treatment of these subjects is clear and practical. We know of no work of its size that commends itself to our judgment so thoroughly as this small compendium of physiology and hygiene.

---

**Hygienic and Sanative Measures for Chronic Catarrhal Inflammation of the Nose, Throat and Ears.** Part I. By THOS. T. RUMBOLD, M. D. St. Louis: Geo. O. Rumbold & Co. 1880.

So much has been written and so many little books published of late about catarrh and its treatment, that a new book on the same subject can scarcely be said to be much needed. Still, in this book the author leaves the trodden path, and takes a new departure in treating especially the hygiene, and also the sanative measures to prevent catarrh, and from this point of view his little work is readable.

---

**The Practitioner's Hand-Book of Treatment on the Principles of Therapeutics.** By J. MILNER FOTHERGILL, M. D. Second American from the second English edition, enlarged. Philadelphia: Henry C. Lea's Son & Co. 1880.

This work differs considerably from other works on therapeutics, being an attempt to explain the rationale of our therapeutic measures on physiological grounds. Physicians are altogether too much in the habit of prescribing for the sick without making it clear for themselves, why they, under such and such conditions, choose such remedies. The author has tried and succeeded in remedying this defect in the medical education and produced a work in which therapeutics, physiology and pathology go hand in hand. We recommend it therefore most heartily to the profession.

---

**Index Catalogue of the Library of the Surgeon General's Office, U. S. Army.** Vol. I. Washington: Government Printing Office. 1880.

This great work has been compiled under the supervision of Dr. J. S. Billings, U. S. A., and reflects the highest honor upon him. Commenced in 1873, the work has been carried on with wonderful industry and persistence. We have here a volume of over 1,000 pages, containing the titles of 9,090 authors, representing 14,429 volumes and pamphlets, 9,000 subject titles and 34,604 titles of articles in periodicals. The magnitude of the work can be estimated by the fact, that this volume of the Index only covers the letter A to Ber, the arrangement of the work being alphabetical. The Index when completed, and we hope there will be no hindrance to the rapid prosecution of the work, will form a respectable library of itself.

---

**The Art of Prolonging Life.** Edited by ERASMUS WILSON, M. D.

Since it is a natural characteristic of the human heart to crave length of life—merely for life's sake, and irrespective of its good or evil condition—this excellent treatise on the subject, from the German of Christopher William Hufeland, and edited by Dr. Wilson, ought to find many interested readers. The simple natural rules laid down for the prolongation of man's earthly existence commend themselves to the common sense of all, the soundness of the ideas being further proved by numerous examples drawn from the history of the past. The original work was published nearly 100 years ago, still it is singular to observe how applicable to the present time is the advice given, and how needful to poor humanity "who know the right, but still the wrong pursue," is this reiteration of much that was already understood, yet daily disregarded.

